# Period shaming behavior among male students in Luang Prabang Province, Lao People’s Democratic Republic: A cross-sectional study

**DOI:** 10.1371/journal.pone.0288145

**Published:** 2023-07-06

**Authors:** Souphalak Inthaphatha, Leyla Isin-Xiong, Viengsakhone Louangpradith, Valee Xiong, Vue Xaitengcha, Alongkone Phengsavanh, Kimihiro Nishino, Nobuyuki Hamajima, Eiko Yamamoto

**Affiliations:** 1 Department of Healthcare Administration, Nagoya University Graduate School of Medicine, Nagoya, Aichi, Japan; 2 Days for Girls International, Luang Prabang City, Luang Prabang Province, Lao People’s Democratic Republic; 3 Department of Healthcare and Rehabilitation, Ministry of Health, Vientiane Capital, Lao People’s Democratic Republic; 4 Faculty of Medicine, University of Health Sciences, Vientiane Capital, Lao People’s Democratic Republic; Zagazig University Faculty of Human Medicine, EGYPT

## Abstract

Period shaming is defined as any negative and/or disrespectful behavior in relation to the menstrual cycle and menstruating girls. It is suggested that period shaming may limit girls’ potential and ability to fully participate in school and community activities. This study aims to examine the prevalence and factors associated with period shaming among male students in Luang Prabang Province, Lao People’s Democratic Republic (Lao PDR). This was a cross-sectional study which was conducted during November 19–27, 2020. This study included 1,232 male students from secondary school grade 9 to 12 in Luang Prabang Province, Lao PDR. Informed consent was obtained from the participants, parents/guardians, and teachers prior to data collection. The data was collected by a self-administered questionnaire. Logistic regression was employed to examine factors associated with period shaming behavior among male students. The mean age of the participants was 16.4 years old. Of all the male students, 18.8% admitted that they had shamed girls during their menstruation at least once. Of those who committed period shaming, they shamed girls some of the times (63.2%). Male students who had consumed alcohol during the last month before the data collection day (AOR = 1.83, 95% CI 1.32–2.55, P<0.001), had heard of menstruation (AOR = 1.76, 95% CI 1.27–2.44, P<0.001), and those who had attended activities/classes about sexual reproductive health (AOR = 1.90, 95% CI 1.29–2.78, P<0.01), were significantly associated with period shaming behavior. In conclusion, a single focus on providing biological health education may not be enough to address menstrual stigmatization and taboos. The school curriculum should integrate other life skill education with reproductive health, such as respect and gender equality, to encourage behavioral changes among male students, to address menstrual stigma and to support and empower girls’ menstrual health at school and in the community.

## Introduction

Menstruation is a natural process, which girls experience on a monthly basis. However, girls’ perception of menstruation can be influenced by how society views menstruation [1], which is often negative (i.e., disgusting and something that needs to be kept a secret) [2]. A qualitative study on boys’ perception of menstruation in India revealed that boys generally perceived menstruation as unclean and dirty. Some boys expressed an extreme negative attitude that water and/or food should not be shared with menstruating girls [3]. Menstrual odor and menstrual leaks are often perceived as strongly inappropriate [4] and girls often have to avoid sitting near other people, especially boys [2, 3]. Such negativities regarding menstruation result in the limitation of resources for girls to manage their menstruation safely and hygienically [4], thus, it reinforces the cycle of stigmatization.

Misinformation and lack of knowledge about menstruation often lead to menstrual myths, cultural taboos, stigma, and shame in the community. Although many countries have integrated menstrual education into the school curriculum [4–7], the contents are mainly focused on biological knowledge and lack of intervention to reduce period stigma, as well as a lack of positive messages to influence the behavior and perception changes [7]. A study in Taiwan revealed that more than half of boys in the study were not interested in learning about menstruation at school, and some boys showed a negative attitude in regard to learning about biology of menstrual cycle at schools [5]. Therefore, boys often receive fragmented information from their families, peers, and the Internet [4, 7].

Although menstruation is a natural process that occurs in female bodies, improving boys’ awareness about menstruation, building good relationships, and encouraging gender respect could combat negative cultural norms and reduce the period stigma. It was reported that men who had menstruating siblings or had romantic relationships with their girlfriends were more likely to have sympathy towards menstruating girls and less likely to engage in period shaming [6]. On the other hand, men who were younger, single, and had less sexual experiences viewed menstruation as an awful matter [8].

According to a recent study in Luang Prabang Province, 77.7% of girls avoided changing pads at school, and 31.8% of girls were absent from school due to menstruation. The school absence might be due to the inadequate toilet facilities that did not support girls in fully participating schools (i.e., there were no gender-separated toilets, and no waste bins in the toilets), therefore, girls avoided using school toilets [9]. Inadequate facilities may impose poor menstrual hygiene practices in girls, and poor hygiene often leads to negative menstrual experiences, including menstrual shaming, particularly, when the toilets are not gender separated. Thus, girls’ menstrual experiences can be influenced by men, including male peers, and male teachers [10]. Information regarding the level of knowledge about puberty and menstruation in male students is limited, especially in Lao PDR. Therefore, this study aims to explore male students’ knowledge on puberty and the prevalence of period shaming at secondary schools and factors associated with such behaviors among male students.

## Materials and methods

### Study setting and study population

This was a cross-sectional study which was conducted between November 19 and 27, 2020, in Luang Prabang Province. Luang Prabang Province is located in the northern part of Lao PDR. The province covers an area of 16,875 km^2^ and has a diverse population of 450,000 people [11]. There are 12 operational districts, and Luang Prabang City is the capital district of the province. According to the Ministry of Education and Sports of Lao PDR, there were 45 public completed secondary schools (grade 6 to 12) in the province, which more than half of the schools are located in Luang Prabang City [12]. In addition, the fiscal year 2018–2019 report revealed there were 49,597 students studying from grade 9 to grade 12 in Luang Prabang Province, and male students accounted for 54% of the total student population in the province [12].

The study sites were sampled with a mixed method (lottery and convenient sampling). First, after obtaining the school list, the schools were divided into schools in the city and schools outside the city. Then, out of 24 public schools in Luang Prabang City, four schools were drawn by lottery sampling from the list. In contrast, the convenient sampling was employed with two schools (two districts) outside Luang Prabang City by taking distance and school accessibility into account. In the end, the study was conducted in six public schools across three districts. There were four schools in Luang Prabang City (Santiphab secondary school, Phanluang secondary school, Pangkham secondary school, and Pasathipatai secondary school), one school in Chomphet district (Chomphet secondary school), and one school in Pakxeng district (Sobjaek secondary school). All male students of grade 9–12 at the six secondary schools who provided written informed consent and those who could secure parental and/or teacher’s consent were included in this study. A total of 1,448 male students met the criteria and consented to answer the questionnaire, however, 216 students were excluded because they did not complete the questionnaire. In the end, 1,232 male students were included in the study.

### Instrument and variables

In this study, period shaming was defined as any negative and/or disrespectful behavior in relation to the menstrual cycle and menstruating girls [13]. A structured questionnaire was developed for self-administration among male students. The questionnaire consisted of three parts: socio-demographic characteristics of participants, menstrual knowledge and behavior about puberty, and menstruation of girl students.

Socio-demographic characteristics were categorized as follows: age (≤16 years old and >16 years old), grade (9, 10, 11 and 12), ethno-linguistics (Lao-Tai, Hmong-Mien and Mon-Khmer), religion (Buddhism, Animism, and Christian), weekly allowance (<50,000LAK per week and ≥50,000LAK per week), residence (live with parents, live with relatives, and stay at the dorm/rental flat), area of school (inside Luang Prabang City and outside Luang Prabang City), and parental education (lower secondary school education or lower and upper secondary school education or higher).

Regarding menstrual and puberty knowledge, there were four multiple choice questions which were used to identify the level of knowledge: Q1) What is menstruation? (bad blood that women shed monthly, normal bleeding from women’s bodies, diseases, other answers and don’t know), Q2) What causes menstruation? (due to hormones, due to the body wants to shed bad blood, diseases, other answers, and don’t know), Q3) Where is the origin of menstrual blood (blood vessel, uterus, abdomen, bladder, birth canal and don’t know), Q4) Which phase of the menstrual cycle is susceptible to pregnancy? The answer choices included: menstrual phase or bleeding phase (day 1–7 of menstrual cycle), follicular phase or egg proliferation phase (day 8–12 of menstrual cycle), ovulation phase (day 14 of menstrual cycle), luteal phase or pre-bleeding phase (day 15–28 of menstrual cycle), and don’t know. Students who could answer three questions or more correctly were categorized as having good menstrual knowledge. Students who could answer two questions or less were categorized as having poor menstrual knowledge. The internal consistency of the level of menstrual knowledge cut-off was good, with Cronbach’s alpha for internal consistency at 0.711. In addition, we asked if the male students have ever heard of menstruation from any sources (yes/no) and whether they have attended (or recalled to attend) activities and/or classroom that provided sexual and reproductive health education (yes/no). For their puberty knowledge, we asked if the male students knew what a wet dream was (yes/no).

In terms of behavior related to menstruation and puberty, we first asked if they had ever had a wet dream experience? (yes/no), age at first wet dream experience (≤14 years old and >14 years old). During the last month, have you consumed any alcohol? (yes/no). During the last month, have you smoked any cigarettes? (yes/no). Regarding period shaming behavior, we asked if they had ever heard of menstruation? (yes/no). Then, have you teased or shamed girls in the past six months when you were aware (witnessed or heard) that the girls were on period? The answer was categorized into yes/no. If boys answered that they committed period shaming, they were asked to answer a probe question about the frequency of period shaming. The answer choices were divided into four choices namely: shaming some of the time, shaming often, shaming most of the time, and shaming all the time.

### Data analysis

The study employed the Statistical Package for Social Sciences, version 28 (IBM SPSS Inc, Armonk, NY, USA) for data analysis. Descriptive analysis was used to understand the frequency and describe the characteristics of the participants. Period shaming behavior was set as an outcome variable. Socio-demographic factors, menstrual knowledge, and behaviors related to puberty were set as determinant factors. Logistic regression analysis was employed to determine the association between the determinants and the outcome. P <0.05 was considered as statistically significant.

### Ethical consideration

The study obtained approval from the National Ethics Committee for Health Research in Lao PDR (No. 066/NECHR). All male students who participated in this study have provided written informed consent regardless of age. In addition, for any participants who were under 18 years old, parental/guardian consent was obtained. In the case where a student stayed in a dorm or in a rental flat, where they had no guardian staying with them, the classroom teacher’s consent was secured for any underage participants. Parent/teacher consent was obtained three days prior to the data collection day.

### Inclusivity in global research

Additional information regarding the ethical, cultural, and scientific considerations specific to inclusivity in global research is included in the S1 Checklist.

## Results

### Socio-demographic characteristics of male students

Of the 1,232 male students who participated in this study, the mean age of the participants was 16.4 years old and the standard deviation of participant’s age is 1.6 years. More than half of the male students were Lao-Tai (56.8%), Buddhist (56.7%), and had a weekly allowance lower than 50,000 LAK (56.9%). The majority of male students lived with their parents (70.9%) and studied at schools in Luang Prabang City (83.6%). There were 638 fathers (51.8%) and 786 mothers (63.8%) of the male students had lower secondary school education or lower (Table 1).

**Table 1:** Socio-demographic characteristics of male students (N = 1,232).

Variables	Total N (%)
Age (M = 16.4, SD = 1.6)	
≤16 years old	665 (54.0)
>16 years old	567 (46.0)
Grade	
Grade 9	144 (11.7)
Grade 10	332 (26.9)
Grade 11	350 (28.4)
Grade 12	406 (33.0)
Ethno-linguistics	
Lao-Tai	700 (56.8)
Hmong-Mien	383 (30.8)
Mon-Khmer	153 (12.4)
Religion	
Buddhism	699 (56.7)
Animism	481 (39.0)
Christian	52 (4.3)
Allowance per week	
<50,000 LAK	701 (56.9)
≥50,000 LAK	531 (43.1)
Residence	
Live with parents	873 (70.9)
Live with relatives	174 (14.1)
Stay at the dorm/rental flat	185 (15.0)
Area of school	
Inside Luang Prabang City	1030 (83.6)
Outside Luang Prabang City	202 (16.4)
Father’s education	
Lower secondary education or lower	638 (51.8)
Higher secondary education or higher	594 (48.2)
Mother’s education	
Lower secondary education or lower	786 (63.8)
Higher secondary education or higher	446 (36.2)

M, mean; SD, standard deviation.

LAK, Lao Kip (1USD = 17,346 LAK, as of October 20, 2022).

### Menstrual knowledge among male students

When inquiring about menstruation, the majority of male students did not understand about menstruation and 88.9% of them had poor menstrual knowledge (Table 2). Most of them selected the answer “I don’t know” for all the questions. However, 26.7% of male students responded that menstruation was ‘bad blood’. Regarding the cause of menstruation, 20.7% of them believed that the cause of menstruation was due to the body that wanted to shed bad blood. A noticeable number of students responded that menstruation came from the birth canal (28.7%), and only 12.2% of students were aware that the ovulation phase is the critical period when women are the most fertile if sexual intercourse occurs. More than half of the male students (58.3%) responded that they have heard of menstruation from any sources. When inquiring about wet dreams, 831 male students (67.5%) could not answer about wet dreams (they either reported that they did not know what a wet dream was or reported that they did but could not accurately define it).

**Table 2:** Knowledge regarding menstruation and puberty among male students (N = 1,232).

Variables	Total N (%)
Q1. What is menstruation?	
Normal bleeding from women’s bodies	373 (30.3)
Bad blood that women shed monthly	329 (26.7)
Diseases	40 (3.2)
Other answers	7 (0.6)
Don’t know	483 (39.2)
Q2. What causes menstruation?	
Hormones	363 (29.5)
Body wanted to shed bad blood	255 (20.7)
Diseases	4 (0.3)
Other answers	19 (1.5)
Don’t know	591 (48.0)
Q3. Origin of menstrual blood	
Uterus	369 (30.0)
Birth canal	353 (28.7)
Blood vessel	6 (0.5)
Abdomen	2 (0.2)
Bladder	15 (1.2)
Don’t know	487 (39.5)
Q4. Which phase of the menstrual cycle is susceptible to get pregnancy?	
Ovulation phase	150 (12.2)
Menstrual phase (bleeding phase)	127 (10.3)
Follicular phase (egg proliferation phase)	112 (9.1)
Luteal phase (pre-bleeding phase)	53 (4.3)
Don’t know	790 (64.1)
Level of menstrual knowledge Q1-Q4^a^	
Poor	1095 (88.9)
Good	137 (11.1)
Have you ever heard of menstruation from any sources?	
Yes	718 (58.3)
No	514 (41.7)
Do you know what a wet dream is?	
Yes	401 (32.5)
No	831 (67.5)

^a^The level of menstrual knowledge was decided by the proportion of correct answers.

### Behaviors related to puberty and period shaming among male students

Table 3 shows the behavior related to puberty and period shaming among male students. More than half of the students (54.1%) responded that they had not had any wet dream experiences. Among those who had one, the average age of experiencing the wet dream was 14 years old. Most students said that they had never participated in any classes or activities that taught about sexual reproductive health (85.9%). There were 480 male students (39.0%) who consumed alcohol, and 8.3% of male students smoked cigarettes, during the last month before the data collection day. Of all the participants, 231 of them (18.8%) admitted that they have shamed girls at least once during the last six months, and 63.2% of those who committed period shaming they shamed girls some of the times.

**Table 3:** Behavior related to puberty and period shaming among male students (N = 1,232).

Variables	Total N (%)
Have you experienced a wet dream?	
Yes	565 (45.9)
No	667 (54.1)
Age at the first wet dream experience	
≤14 years old	337 (59.6)
>14 years old	228 (40.4)
Have you attended activities/classes that taught about sexual reproductive health?	
Yes	174 (14.1)
No	1058 (85.9)
Have you consumed any alcohol during the last month?	
Yes	480 (39.0)
No	752 (61.0)
Have you smoked cigarettes during the last month?	
Yes	102 (8.3)
No	1130 (91.7)
Have you ever shamed or teased girls when they had menstruation?	
Yes	231 (18.8)
No	1001 (81.2)
Frequency of committing period shaming	
Shame some of the times	146 (63.2)
Shame often	58 (25.1)
Shame most of the times	9 (3.9)
Shame all the times	18 (7.8)

### Factors associated with period shaming among male students

Table 4 shows the analysis of logistic regression of key factors associated with period shaming behavior among male students. As a result, male students who had consumed alcohol during the last month before the data collection day (AOR = 1.83, 95% CI 1.32–2.55, P<0.001) were more likely to shame girls about menstruation. In addition, male students who had heard of menstruation from any sources (AOR = 1.76, 95% CI 1.27–2.44, P<0.001), and those who recalled that they had attended activities/classes about sexual reproductive health (AOR = 1.90, 95% CI 1.29–2.78, P<0.01), were significantly associated with period shaming behavior than those who did not heard about menstruation or those who did not recall attending sexual reproductive health class. The smoking behavior showed significant associations to period teasing in the binary logistic analysis; however, it depreciated after being adjusted in the multiple logistic regression model.

**Table 4:** Factors associated with period shaming among male students (N = 1,232).

Variables	Period shaming behavior towards girls		OR 95% CI	AOR 95% CI
	Yes n (%)	No n (%)		
Age				
≤16 years old	119 (17.9)	546 (82.1)	1 (reference)	1 (reference)
>16 years old	112 (19.8)	455 (80.2)	1.14 (0.86–1.52)	0.92 (0.60–1.41)
Grade				
Grade 9	26 (18.1)	118 (81.9)	1 (reference)	1 (reference)
Grade 10	52 (15.7)	280 (84.3)	0.86 (0.51–1.44)	0.75 (0.43–1.29)
Grade 11	74 (21.1)	276 (78.9)	1.25 (0.76–2.05)	1.02 (0.58–1.78)
Grade 12	79 (19.5)	327 (80.5)	1.13 (0.69–1.84)	0.83 (0.43–1.60)
Ethno-linguistics				
Lao-Tai	133 (19.0)	567 (81.0)	1 (reference)	1 (reference)
Hmong-Mien	78 (20.6)	301 (79.4)	1.10 (0.81–1.50)	1.04 (0.68–1.58)
Mon-Khmer	20 (13.1)	133 (86.9)	0.64 (0.38–1.06)	**0.51 (0.29–0.91)** *
Allowance per week				
<50,000 LAK	126 (18.0)	575 (82.0)	1 (reference)	1 (reference)
≥50,000 LAK	105 (19.8)	426 (80.2)	1.12 (0.84–1.49)	0.99 (0.71–1.37)
Residence				
Live with parents	158 (18.1)	715 (81.9)	1 (reference)	1 (reference)
Live with relatives	37 (21.3)	137 (78.7)	1.24 (0.83–1.85)	1.28 (0.82–1.98)
Stay at the dorm /rental apartment	36 (19.5)	149 (80.5)	1.10 (0.74–1.65)	1.13 (0.70–1.82)
Area of school				
Outside Luang Prabang City	38 (18.8)	164 (81.2)	1 (reference)	1 (reference)
Inside Luang Prabang City	193 (18.7)	837 (81.3)	1.00 (0.68–1.46)	1.11 (0.71–1.73)
Father’s education				
Lower secondary education or lower	124 (19.4)	514 (80.6)	1 (reference)	1 (reference)
Higher secondary education or higher	107 (18.0)	487 (82.0)	0.91 (0.69–1.22)	0.99 (0.66–1.48)
Mother’s education				
Lower secondary education or lower	157 (20.0)	629 (80.0)	1 (reference)	1 (reference)
Higher secondary education or higher	74 (16.6)	372 (83.4)	0.80 (0.59–1.08)	0.70 (0.46–1.07)
Level of menstrual knowledge				
Poor	197 (18.0)	898 (82.0)	1 (reference)	1 (reference)
Good	34 (24.8)	103 (75.2)	1.51 (0.99–2.28)	1.32 (0.85–2.05)
Have you experienced wet dream?				
No	115 (17.2)	552 (82.8)	1 (reference)	1 (reference)
Yes	116 (20.5)	449 (79.5)	1.24 (0.93–1.65)	1.01 (0.74–1.39)
Have you consumed any alcohol during the last month?				
No	115 (15.3)	637 (84.7)	1 (reference)	1 (reference)
Yes	116 (24.2)	364 (75.8)	**1.77 (1.32–2.36)** ***	**1.83 (1.32–2.55)** ***
Have you smoked any cigarette during the last month?				
No	202 (17.9)	928 (82.1)	1 (reference)	1 (reference)
Yes	29 (28.4)	73 (71.6)	**1.83 (1.16–2.88)** *	1.42 (0.86–2.34)
Have you ever heard of menstruation from any sources?				
No	71 (13.8)	443 (86.2)	1 (reference)	1 (reference)
Yes	160 (22.3)	558 (77.7)	**1.79 (1.32–2.43)** ***	**1.76 (1.27–2.44)** ***
Have you attended activities/classes about sexual reproductive health?				
No	180 (17.0)	878 (83.0)	1 (reference)	1 (reference)
Yes	51 (29.3)	123 (70.7)	**2.02 (1.41–2.91)** ***	**1.90 (1.29–2.78)** **

*P<0.05,

**P<0.01,

***P<0.001

OR, odds ratio; AOR, adjusted odds ratio; CI, confidence interval; LAK, Lao Kip (1USD = 17,346 LAK, as of October 20, 2022).

## Discussion

This study provided an insight into period shaming and the factors that contributed to such behavior among male students in Luang Prabang Province of Lao PDR. In our study, we found that 18.8% of male students shamed girl students when they were aware that the girls had their periods. Although the results in our study are consistent with the previously referenced study in Northern Tanzania, where 18% of boys engaged in period teasing in schools [4], the result in this study may be underreported. Given that 145 male students surveyed did not report their period shaming behavior, thus, they were excluded from this study. Male students might be unwilling to report their own behaviors even though there were no names written on the questionnaire to indicate them. The withholding behavior about period shaming might be due to the fear of being judged. The qualitative study in India has revealed that among 85 male students who believed menstruation was awful, most of them did not mention about committing period shaming directly and only one of them openly indicated that menstruating girls should not be shamed [3].

Providing biological-focused health education is not enough to combat period shaming and stigma [4]. In our study, male students, who had heard of menstruation and those who had attended sexual reproductive health classes, were more likely to shame girls during their menstruation. Male students might receive fragments of menstrual information from many sources, including the school curriculum, friends and family members (their sisters’ menarche) [4, 5, 14]. In Lao PDR, menstruation and reproductive health education are briefly included in formal education; however, the content is fragmented and vague. Contents in the Natural Sciences Textbook of secondary school grade 9, including puberty changes, sexual reproduction, family planning, and sexually transmitted diseases, mainly focus on biological information [7]. Despite the result in this study which suggested that male students who have attended the education class had significantly good biological-menstrual knowledge, they are also more likely to shame girls during menstruation, although the significance level depreciates after adjustment. The result of this study emphasizes the importance of holistic menstrual health education that goes beyond biological content. The school curriculum should integrate other life skill education, such as respect and gender equality, with reproductive health, to encourage behavioral changes among male students to support and empower girls’ menstrual health at school [13].

Interestingly, in our study, male students who had consumed alcohol during the last month before data collection were more likely to shame girls about their menstruation than those who did not consume any. The result in our study is consistent with the study in Chile, which revealed a strong association between school bullies and alcohol consumption [15]. In addition, the research from Canada suggested that being a bully at the baseline was associated with initiating binge drinking within the following two years [16]. Although the explanation of the relationship between alcohol and bullying is unclear [16], and it could be influenced by many other factors, such as parents, peers, and peer pressure [17, 18]. Nonetheless, alcohol often leads to negative behavior, including drinking and driving, risky sexual behavior, and violence [19–21]. Laws and health policies on alcohol consumption among underage groups should be strengthened and strictly enforced to prevent and reduce underage drinking.

Despite being conducted in only Luang Prabang Province, our study consisted of a relatively strong ethnic diversity of participants (not a majority of Lao-Tai) [22]. However, this study has some limitations. First, as the study used a self-report questionnaire, some students may be reluctant to answer truthfully, and recall biases may have occurred. Secondly, the current study focuses mainly on the quantitative observational questionnaire study; thus, the information behind some causal relationships is unable to be disclosed. Finally, our study was conducted among secondary male students in Luang Prabang Province, where some beliefs and characteristics may be unique to the province. Additional studies that include an increased and diverse number of provinces will enhance the data and findings for a better understanding of this topic nationally.

## Conclusion

A noticeable proportion of male students (18.8%) in secondary schools shamed girls during their menstruation. Factors associated with period shaming behavior were those who had heard of menstruation from any sources, those who recalled they had participated in sexual reproductive health class, and those who had consumed alcohol during the last month before data collection. A single focus on providing biological health education/information may not be enough to address this issue. The school curriculum should integrate other life skill education, such as respect and gender equality, with reproductive health, to encourage behavioral changes among male students to support and empower girls’ menstrual health at school.

## Supporting information

S1 Checklist Inclusivity in global research. (DOCX)
